# The Role of Brain Frailty in Stroke Outcomes: A Systematic Review

**DOI:** 10.3390/neurolint18080147

**Published:** 2026-08-03

**Authors:** Hussain Almohammed, Husna Irfan Thalib, Samiya Khanam, Kawthar Faisal Kushara, Wejdan Ahmed Aldawsari, Hala Omar Algazzawi, Rimas Warid Aljuaid, Mohammed Ibrahim Almuhaysin, Hams Akram Alharbi, Alia Alokley

**Affiliations:** 1 College of Medicine, King Faisal University, Al Ahsa 31982, Saudi Arabia; rm13hf2002@gmail.com; 2 General Medicine Practice Program, Batterjee Medical College, Jeddah 21442, Saudi Arabia; husnairfan2905@gmail.com (H.I.T.); halaalgazzawi@gmail.com (H.O.A.); 3 College of Medicine, King Abdulaziz University, Jeddah 21589, Saudi Arabia; kawthar3344552@gmail.com (K.F.K.); hamsalahmadii@gmail.com (H.A.A.); 4 College of Medicine, Tabuk University, Tabuk 47918, Saudi Arabia; drwejdanahmed@gmail.com; 5 College of Medicine, Taif University, Taif 21944, Saudi Arabia; rahood21@gmail.com; 6 College of Medicine, University of Groningen, 9712 Groningen, The Netherlands; mohmed_ibr@hotmail.com; 7 Departments of Clinical Neuroscience, College of Medicine, King Faisal University, Al-Ahsa 31982, Saudi Arabia

**Keywords:** brain frailty, cerebral small vessel disease, stroke, cognitive impairment, functional outcome

## Abstract

Background and objectives: Cerebrovascular strokes and transient ischemic attacks (TIAs) remain leading causes of global mortality and long-term disability. Emerging evidence suggests that pre-existing structural brain frailty—driven by chronic cerebral small vessel disease (CSVD)—critically limits neuroplastic compensation and determines overall recovery potential. This systematic review aims to synthesize current evidence evaluating brain frailty as an independent determinant of functional and cognitive outcomes. Methods: Following PRISMA and Cochrane SWiM guidelines, a comprehensive literature search was conducted across PubMed, Web of Science, Embase, and Scopus. Methodological quality was evaluated using the Newcastle–Ottawa Scale (NOS). Due to anticipated clinical and methodological heterogeneity, data were synthesized via a structured narrative approach. A total of 15 studies comprising more than 18,000 participants met the inclusion criteria, including prospective cohorts, retrospective cohorts, registry analyses, pooled cohort studies, and post hoc analyses of randomized controlled trials. Results: Structural disconnection burden emerged as one of the strongest predictors of post-stroke cognitive impairment, demonstrating remarkably large effect sizes at both 6 months (OR 9.96, 95% CI 2.21–52.40) and 36 months (OR 12.27, 95% CI 2.80–63.40). Additionally, blood–brain barrier (BBB) leakage was significantly associated with longitudinal cognitive decline (β −3.62 to −0.16, *p* < 0.001). Pre-existing brain frailty was also consistently associated with poorer functional recovery following acute ischemic stroke, even among patients undergoing advanced reperfusion strategies such as endovascular thrombectomy or intravenous thrombolysis. Conclusions: Brain frailty should be integrated into clinical prognostic frameworks as a key marker for predicting long-term cognitive function and functional independence in stroke patients. However, the substantial methodological and clinical heterogeneity observed across the included literature must be acknowledged as a key limitation of the current evidence base. Large-scale, prospective, multicenter longitudinal studies are required to better standardize brain frailty metrics and validate their utility in clinical practice.

## 1. Introduction

Cerebrovascular strokes have persistently remained one of the leading causes of long-term disability and mortality worldwide for decades. A landmark study conducted in 2016 estimated an annual mortality rate of 5.5 million, alongside 116.4 million cases of long-term disability attributed to stroke [1]. Ischemic strokes contribute to approximately 62.4% of all stroke incidents, whereas intracerebral hemorrhages (ICH) and subarachnoid hemorrhages constitute 27.9% and 9.7% of cases, respectively [2]. The substantial increase in stroke prevalence over the last two decades has been strongly associated with rapidly rising modifiable risk factors, including elevated body mass index (BMI), hyperlipidemia, and hypertension [3,4]. Conversely, a transient ischemic attack (TIA) is defined as a transient hypoperfusion of brain tissue lacking visible infarction on brain magnetic resonance imaging (MRI) and featuring a 24-h symptom resolution window [5]. Although TIAs are clinically transient, studies have demonstrated a non-negligible risk of ischemic stroke incidence within the three-month post-TIA window—ranging from 3% to 20%—along with an elevated 5-year mortality rate of 18.6% [6].

Stroke recovery outcomes are highly heterogeneous and dynamic, characterized by varying treatment responses and prognostic findings across diverse baseline profiles and severity strata. Distinct recovery trajectories have been observed even among patients who present with similar infarct sizes on neuroimaging, follow identical treatment guidelines, and achieve successful reperfusion and recanalization. These disparate recovery patterns are thought to be influenced by both the severity of the acute vascular insult and the underlying functional and structural integrity of the brain parenchyma [7,8].

Many contemporary stroke prognostic frameworks rely primarily on acute severity scales within clinical contexts. The National Institutes of Health Stroke Scale (NIHSS) captures neurological deficits at presentation—encompassing both motor and non-motor stroke dysfunctions—with impairment scores ranging from 0 to 42. The reliability and validity of the NIHSS can be enhanced when used in combination with other assessment tools, such as the Motor Assessment Scale (MAS) [8]. Other clinical prognostic factors and scoring systems include MRI- and CT-defined infarct burden, the Stroke Prognosis Instrument I and II [SPI-I/II]—which stratifies stroke patients based on a two-year prognostic window for mortality or recurrent stroke [9,10]—and the Totaled Health Risks in Vascular Events (THRIVE) score, which incorporates three baseline comorbidities into its prognostic stratification [11].

In comparison to the aforementioned severity and risk-stratification tools, the Modified Rankin Scale (mRS) is a functional outcome scale designed to measure global disability and dependence following a cerebrovascular event. The mRS score ranges from 0 to 6 across six distinct disability categories [12,13]. Over the years, the mRS has gained widespread popularity and adoption, becoming the primary endpoint in stroke research; however, its utility was historically limited by high inter-observer variability, an issue later addressed by the development of the simplified mRS questionnaire (smRSq) [13].

Despite the diverse clinical assessment objectives of the NIHSS and mRS, both scales remain fundamentally outcome-oriented and fail to measure underlying intrinsic brain resilience and structure. Consequently, current prognostic models incompletely capture the biological substrate that ultimately determines recovery potential following a cerebrovascular injury [14,15].

Cerebral small vessel disease (CSVD) constitutes a spectrum of intracranial vascular conditions that underlie ischemic strokes, TIAs, and intracerebral hemorrhages. The pathophysiology of CSVD is driven by the dysfunction of perforating arterioles, capillaries, and venules via arteriolosclerosis, cerebral amyloid angiopathy, and other vascular pathologies. These changes lead to decreased cerebral perfusion, impaired autoregulation, and increased blood–brain barrier (BBB) permeability, collectively culminating in brain and cognitive dysfunction [16,17,18].

Given the clinically silent nature of this disease, diagnosis is primarily achieved through neuroimaging screening methods. Neuroimaging markers of CSVD include parenchymal microbleeds, lacunar infarcts, enlarged perivascular spaces, white matter hyperintensities (WMH), and brain atrophy [18]. WMHs represent the most reliable marker of chronic microvascular injury, as white matter and watershed areas are the cerebral regions most vulnerable to hypoperfusion. Lacunar infarcts account for approximately 25% of all acute ischemic strokes, whereas subcortical infarcts are attributed to ischemia within a region supplied by a single perforating artery. Radiological findings of CSVD show strong associations with hypertension, diabetes, advanced age, smoking, and dyslipidemia. These vascular risk factors can acutely occlude larger cerebral arteries—leading to overt strokes—or cause chronic hypoperfusion and deep white matter microinfarctions, thereby imparting an inherent, chronic vulnerability and frailty to the brain tissue [18,19].

Brain atrophy serves as an end-stage cerebral pathology that reflects cumulative injury. This loss of brain parenchymal volume is indicative of underlying neuronal loss, synaptic degeneration, and large-scale network disruption. Consequently, brain atrophy, concurrently with WMHs, is substantially associated with accelerated cognitive decline, gait disturbances, and dementia symptoms [20,21,22].

Brain frailty is characterized as a state of reduced brain reserve in which susceptibility to, and risk from, cerebrovascular injury are dictated more by pre-existing limitations in structural integrity than by the severity of the acute vascular lesion itself. Although strong associations have been established between WMHs and conventional risk factors, the persistent causative variability of WMHs points to additional influences, such as genetic predisposition, chronic inflammation, and intrinsic processes of brain aging. Over time, these mechanisms contribute to the progressive disruption of white matter efficiency, thereby limiting neuroplastic compensation and rendering patients susceptible to poorer functional outcomes following cerebrovascular events [23].

In ischemic stroke, prognostic outcomes are conventionally attributed to infarct volume, infarct location, and the success of intravenous thrombolytic reperfusion therapy. However, a cohort study conducted in 2025 [24] provided clinical evidence of poorer functional outcomes—measured by the mRS at a 90-day follow-up—in post-ischemic stroke patients who had undergone successful reperfusion therapy but possessed a background of pre-existing brain frailty (specifically WMHs, lacunar infarcts, and brain atrophy on MRI). Notably, this negative impact was independent of established, conventional prognostic measures [24,25].

In intracerebral hemorrhage, CSVD plays a crucial role in both the pathogenesis and the outcome of spontaneous intracerebral hemorrhage (SICH). Chronic small vessel pathology, manifested by WMHs, microaneurysms, and cerebral microbleeds, increases a patient’s susceptibility to arterial rupture, hematoma expansion, and perihematomal injury. A retrospective study of 1223 patients with brain frailty demonstrated decreased functional outcomes and higher mortality rates following ICH, independent of hematoma volume and location, thereby influencing both short- and long-term complications [26,27].

In transient ischemic attacks, the underlying brain pathology is frequently underestimated due to the absence of visible infarction on standard neuroimaging. However, patients with underlying cerebrovascular disease and brain frailty face a significantly increased risk of subsequent ischemic strokes and cognitive decline following an initial TIA. This evidence supports the interpretation of a TIA as a manifestation of chronic cerebrovascular pathology rather than a purely transient, isolated event.

Across ischemic stroke, ICH, and TIA, a consistent pattern emerges: structural brain integrity plays a central role in determining both the susceptibility to cerebrovascular events and subsequent recovery potential, extending far beyond acute lesion characteristics alone [28,29].

Evidence linking cerebral small vessel disease and brain frailty to stroke outcomes remains fragmented across separate studies of ischemic stroke, intracerebral hemorrhage, and TIA. Furthermore, these fields are often limited by inconsistent imaging definitions and heterogeneous outcome measures. Most existing studies assess single isolated markers rather than an integrated measure of neurovascular reserve. The aim of this review is to synthesize current evidence across these distinct clinical phenotypes and to evaluate brain frailty as an independent determinant of functional and cognitive outcomes.

## 2. Methodology

This systematic review follows the guidelines of the Preferred Reporting Items for Systematic Reviews and Meta-Analyses (PRISMA), so we reproduce and present the chosen studies [30]. The protocol of the study was submitted in the International Prospective Register of Systematic Reviews (PROSPERO) under registration number CRD420261362715. Due to the type of the study, ethical approval from an institutional research ethics board was not required.

### 2.1. The Search Strategy

A comprehensive literature search across (PubMed, Web of Science, Embase, and Scopus) was performed to extract all the relevant articles from inception until April 2026 by using those keywords in the search engine: (“brain frailty” OR “cerebral frailty” OR “brain vulnerability” OR “cognitive frailty” OR “frailty” OR “frailty syndrome” OR “clinical frailty” OR “clinical frailty scale” OR “frail” OR “pre-frailty” OR “prefrailty”) AND (“stroke” OR “acute ischemic stroke” OR “AIS” OR “ischemic stroke” OR “ischaemic stroke” OR “hemorrhagic stroke” OR “haemorrhagic stroke” OR “intracerebral hemorrhage” OR “intracerebral haemorrhage” OR “ICH” OR “cerebrovascular disease” OR “cerebrovascular accident” OR “CVA” OR “TIA” OR “transient ischemic attack” OR “reperfusion therapy” OR “reperfusion treatment” OR “TPA” OR “tissue plasminogen activator” OR “mechanical thrombectomy” OR “thrombolytics”) AND (“neuroimaging” OR “neuroimaging markers” OR “brain imaging” OR “MRI” OR “magnetic resonance imaging” OR “CT” OR “computed tomography” OR “white matter hyperintensities” OR “WMH” OR “leukoaraiosis” OR “cerebral atrophy” OR “brain atrophy” OR “cortical atrophy” OR “subcortical atrophy” OR “small vessel disease” OR “cerebral small vessel disease” OR “SVD” OR “lacune” OR “lacunes” OR “microbleed” OR “microbleeds” OR “cerebral microbleeds” OR “enlarged perivascular spaces”) AND (“outcome” OR “outcomes” OR “prognosis” OR “prognostic” OR “mortality” OR “death” OR “survival” OR “functional outcome” OR “functional recovery” OR “disability” OR “dependence” OR “modified Rankin Scale” OR “mRS” OR “Functional Independence Measure” OR “FIM” OR “cognitive impairment” OR “cognitive decline” OR “neurological outcome”) AND (“randomized controlled trial” OR “randomized controlled trial” OR “RCT” OR “clinical trial” OR “controlled clinical trial” OR “cohort study” OR “prospective cohort” OR “retrospective cohort” OR “longitudinal study” OR “observational study” OR “observational” OR “comparative study”). The detailed search assessment for each included study is provided in the Supplementary Material (Table S1). To ensure completeness of the search strategy, the retrieved studies were cross-checked against landmark publications identified from reference lists and expert knowledge. No additional eligible studies were identified through this process.

### 2.2. Study Selection

We have selected studies based on predefined inclusion and exclusion criteria. Inclusion criteria are as follows: adult patients who are 18 or older with a diagnosis of a TIA, ischemic or hemorrhagic stroke, regardless of whether they have received reperfusion therapy or not. Eligible studies were required to include at least one neuroimaging-based marker reflecting brain frailty (e.g., white matter hyperintensities, cerebral atrophy, lacunes, chronic infarcts, structural disconnection, or blood–brain barrier dysfunction). Studies evaluating systemic or physical frailty alone, without neuroimaging assessment of the brain, were excluded. Studies assessing both systemic frailty and neuroimaging-defined brain frailty were eligible only when imaging-derived brain frailty markers were separately evaluated or incorporated into the reported analyses.

Additionally, studies must report functional outcomes—using the mRS or the Functional Independence Measure (FIM) at both discharge and 90-day follow-up—or cognitive outcomes—using the Montreal Cognitive Assessment (MoCA) post-stroke. Study types that were included are RCTs and observational cohort studies, both retrospective and prospective, and only studies that were written in English were considered. We have excluded study types such as case reports, case–control studies, reviews, editorials, or conference abstracts. Studies that did not mention brain frailty assessments or report their outcomes were also excluded.

### 2.3. Screening

Screening was performed by two reviewers, who first independently screened titles and abstracts using the Rayyan web tool using the predefined inclusion and exclusion criteria [31]. Studies that met the criteria were reviewed in full text by the same reviewers. Lastly, reviewers discussed among themselves any disagreements, if there were any.

### 2.4. Data Extraction

One reviewer independently extracted data through a pilot extraction that was developed using Excel. For each study used, the reviewer extracted identifier variables, such as (name of the first author, year of publication, country of publication, the journal, and the study title). Additionally, study characteristics data were extracted, including (study design/setting, sample size, duration of the study, inclusion and exclusion criteria used in the study). Furthermore, participant characteristics data were extracted to assess baseline comparability, such as mean/median age, the range of ages among participants, stroke type (ischemic/hemorrhagic), stroke severity, and comorbidities such as hypertension, diabetes, and atrial fibrillation. Finally, the reviewer extracted data on frailty assessments (including imaging tools, frailty scales, and scores) and on functional and cognitive outcomes from discharge to 90 days post-stroke. In addition, the completed PRISMA checklist is provided as a Supplementary File (Table S2).

### 2.5. Risk of Bias Assessments

The quality of the studies was assessed using the Newcastle–Ottawa Scale (NOS). NOS assesses cohort studies; it evaluates the selection of the participants, comparability of the study groups, and the outcome assessments. The higher the NOS score, the lower the risk of bias [32].

### 2.6. Outcomes Measures

The primary outcome of this systematic review is to identify whether brain frailty can serve as a reliable predictor of poor functional and cognitive outcomes in stroke patients. The secondary outcomes include differentiation of the impact of brain frailty between ischemic and hemorrhagic stroke. Also, assess the relationship between brain frailty markers and functional outcomes at both discharge and 90 days post-stroke. Lastly, to assess relationships between brain frailty markers and cognitive outcomes post-insult. For the purpose of this review, brain frailty is operationally defined as a state of reduced structural and functional brain reserve resulting from the cumulative burden of chronic cerebrovascular injury, neurodegeneration, and age-related cerebral changes that increase vulnerability to poor outcomes after stroke. Importantly, brain frailty should not be considered synonymous with cerebral small vessel disease (CSVD). Rather, CSVD represents one major contributor to brain frailty through markers such as white matter hyperintensities, lacunes, and cerebral microbleeds. In contrast, neurodegenerative changes (e.g., cortical, subcortical or medial temporal atrophy), network disruption (e.g., structural disconnection burden), and physiological vascular dysfunction (e.g., blood–brain barrier leakage) represent additional complementary domains that collectively reflect reduced brain reserve.

### 2.7. Data Analysis

A formal meta-analysis was considered; however, due to the small number of included studies and methodological heterogeneity, a meta-analysis was not feasible. If sufficient homogeneity had been present across study populations, interventions, and outcome measures, a random-effects model would have been applied. Statistical heterogeneity would have been assessed using the I^2^ statistic, with values greater than 50% indicating substantial heterogeneity. In the absence of adequate homogeneity, findings were synthesized narratively. Following a structured synthesis approach, in line with Cochrane SwiM (Synthesis Without Meta-analysis) guidance, both descriptive and comparative analyses were conducted. Data were systematically extracted from the included studies, including study characteristics, study design, participant demographics, stroke subtype (ischemic or hemorrhagic), brain frailty assessment methods, and reported functional and cognitive outcomes at discharge and 90 days post-stroke or longer-term follow-up, and cognitive outcomes measured at any validated post-stroke follow-up interval. This facilitated structured cross-study comparison. Where available, associations between brain frailty and outcomes were descriptively compared using reported effect estimates, confidence intervals, and statistical significance measures. Given the limited number of studies and variability in outcome reporting, a vote-counting approach was not formally applied; instead, evidence was synthesized narratively across comparable outcome domains.

## 3. Results

**Figure 1:**
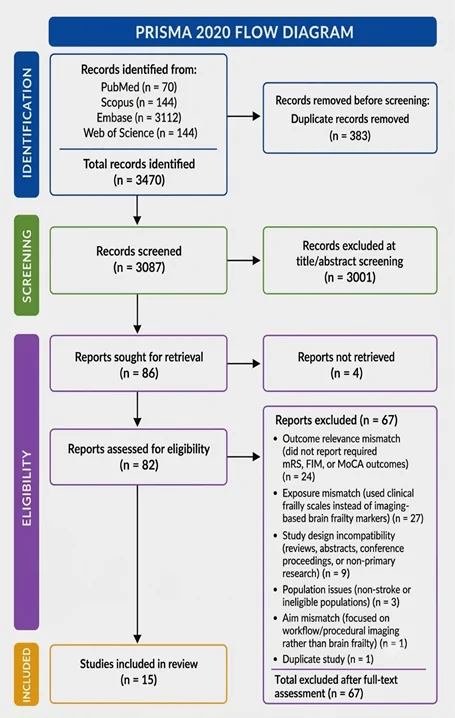
PRISMA flowchart describing the search strategy.

The literature search identified a total of 3470 records from PubMed (*n* = 70), Scopus (n = 144), Embase (n = 3112), and Web of Science (n = 144). After removing 383 duplicate records, 3087 unique records underwent title and abstract screening by two independent reviewers, of which 3001 were excluded. A total of 86 full-text articles were sought for retrieval, of which 4 could not be obtained. Subsequently, 82 full-text articles were assessed for eligibility, with 67 excluded due to outcome relevance mismatch (n = 24), exposure mismatch (n = 27), study design incompatibility (n = 9), population issues (n = 3), aim mismatch (n = 3), and duplicate study (n = 1). Ultimately, 15 studies [24,25,33,34,35,36,37,38,39,40,41,42,43,44,45] met the inclusion criteria and were included in the systematic review (Figure 1).

### 3.1. Study Characteristics

A total of 15 studies were included in the systematic review, comprising prospective cohorts, retrospective cohorts, registry analyses, pooled cohort studies, and post hoc analyses of randomized controlled trials (Table 1). Collectively, these studies evaluated more than 18,000 patients with ischemic stroke or TIA. Sample sizes ranged from 105 participants in the study by Lopes et al. [35] to 8918 participants in the nationwide registry analysis conducted by Gwak et al. [38] (Table 1). The studies were conducted across diverse geographical regions, including Europe, North America, Asia, and multinational cohorts, thereby enhancing the generalizability of the findings. The mean age of participants ranged from 63 to 74 years, reflecting the predominance of older adults in stroke populations. Female representation varied between 31% and 54%, although several studies did not report sex-specific distributions. Follow-up duration ranged from 90 days to 36 months, with most studies assessing outcomes within the first three months after stroke, while several investigations evaluated longer-term cognitive trajectories extending beyond one year (Table 1). Several included studies evaluated long-term cognitive outcomes extending beyond 90 days (up to 36 months). These studies were retained because the review aimed to synthesize both short- and long-term prognostic implications of imaging-defined brain frailty.

Substantial heterogeneity was observed in the operationalization of brain frailty. WMH burden represented the most frequently assessed marker and was evaluated in multiple studies including those by Hong et al. [33], Gwak et al. [38], Mechtouff et al. [41], Coenen et al. [37], and Griessenauer et al. [44]. Other studies adopted multidimensional brain frailty constructs incorporating cerebral atrophy, chronic infarcts, lacunes, leukoaraiosis, hippocampal atrophy, medial temporal atrophy, and strategic lesion burden. Neuroimaging modalities included MRI, CT, non-contrast CT (NCCT), CT angiography (CTA), and dynamic contrast-enhanced MRI (DCE-MRI), reflecting the wide range of imaging approaches used to characterize structural brain vulnerability. Baseline stroke severity, as assessed by the NIHSS, ranged from mild deficits (NIHSS approximately 1 to 3 in minor stroke/TIA cohorts such as Lopes et al. [35] and Sivakumar et al. [42] to severe neurological impairment NIHSS approximately 14 to 19 in cohorts such as Zilioli et al. [36] and Tschirret et al. [40], with most EVT and AIS cohorts clustering in the moderate range (approximately NIHSS 3 to 6), indicating substantial heterogeneity in initial stroke severity across studies (Table 1).

### 3.2. Brain Frailty Assessment Methods

Considerable methodological heterogeneity was observed in the assessment of brain frailty across included studies (Table 2). Several investigations focused on individual neuroimaging markers, particularly WMH burden, quantified either through automated volumetric segmentation or visual rating scales such as the Fazekas score. Other studies employed composite brain frailty scores integrating multiple structural markers, including cerebral atrophy, chronic infarcts, lacunes, and leukoaraiosis. Rowan et al. [25] utilized a CT-based brain frailty score ranging from 0 to 3, incorporating leukoaraiosis, cerebral atrophy, and old infarcts. Similarly, Loewen et al. [24] employed a multidimensional CT-based framework integrating Fazekas WMH scores, global cortical atrophy (GCA), central cerebral atrophy ratios, and chronic infarcts or lacunes. Zilioli et al. [36] used a combination of validated visual rating scales including medial temporal atrophy (MTA), global cortical atrophy-frontal (GCA-F), Koedam posterior atrophy scores, and Fazekas WMH grading.

Other studies explored more specialized markers of structural vulnerability. Lopes et al. [35] investigated structural disconnection burden using a Bayesian latent Dirichlet allocation model, whereas Coenen et al. [37] examined strategic WMH tract burden through MRI-based tract mapping. Wardlaw et al. evaluated BBB leakage using DCE-MRI permeability mapping, providing a functional marker of cerebral vulnerability rather than a conventional structural marker of injury. Collectively, these findings demonstrate that brain frailty encompasses a broad spectrum of neuroimaging abnormalities reflecting cumulative cerebral aging, small vessel disease, neurodegeneration, and prior cerebrovascular injury (Table 2). As shown in Table 3, brain frailty has been operationalized using four major domains: chronic cerebral small vessel disease burden, neurodegenerative changes, network injury, and physiological vascular dysfunction, each represented by distinct neuroimaging biomarkers across the included studies.

### 3.3. Outcomes Evaluated

The primary outcomes assessed across studies are summarized in Table 3. Functional outcomes were the most frequently reported endpoints and were commonly measured using the mRS, neurological recovery indices, or thrombectomy-specific outcome measures. Seven studies primarily focused on functional recovery following ischemic stroke or EVT. Cognitive outcomes represented the second major outcome domain. These included post-stroke cognitive impairment (PSCI), major or minor neurocognitive disorder, cognitive decline, and overall cognitive performance. Several studies specifically investigated the relationship between imaging-defined brain frailty and subsequent neurocognitive outcomes, highlighting the growing recognition of cognitive sequelae as critical determinants of post-stroke prognosis. Additional outcomes included futile recanalization following EVT, early neurological deterioration, and longitudinal changes in NIHSS scores (Table 4).

### 3.4. Association Between Brain Frailty and Cognitive and Functional Outcomes

A particularly strong and consistent relationship was observed between brain frailty markers and post-stroke cognitive outcomes (Table 5). Taylor-Rowan et al. [25] demonstrated that increasing brain frailty significantly elevated the risk of both cognitive impairment and broader frailty-related outcomes, with adjusted odds ratios ranging from 1.64 to 1.93 and a hazard ratio reaching 1.84, underscoring a dose–response relationship between cumulative brain vulnerability and neurocognitive decline. Similarly, Munthe-Kaas et al. [34] reported that medial temporal atrophy independently predicted major neurocognitive disorder at 3 months post-stroke (adjusted OR 3.09), indicating more than a threefold increase in risk associated with focal neurodegenerative structural changes.

Even more pronounced effects were observed in studies assessing advanced imaging-derived markers of network disruption and small vessel disease burden. Lopes et al. [35] demonstrated that structural disconnection burden was one of the strongest predictors of post-stroke cognitive impairment, with very large effect sizes at both 6 months (OR 9.96, 95% CI 2.21–52.40) and 36 months (OR 12.27, 95% CI 2.80–63.40), suggesting sustained long-term cognitive vulnerability. Similarly, Sivakumar et al. [42] reported a nearly tenfold increase in persistent cognitive impairment among patients with higher burdens of MRI-defined small vessel disease markers (OR 9.42, 95% CI 1.96–45.37), reinforcing the critical role of cerebrovascular pathology in post-stroke cognitive trajectories.

Supporting these findings, Coenen et al. [37] identified a significant association between strategic WMH tract involvement and post-stroke cognitive impairment, highlighting the importance of lesion location rather than burden alone. Furthermore, Wardlaw et al. [39] demonstrated that BBB leakage was significantly associated with longitudinal cognitive decline (β −3.62 to −0.16, *p* < 0.001), providing mechanistic evidence that vascular dysfunction contributes to progressive neurodegeneration after stroke. Collectively, these studies consistently indicate that structural brain frailty, small vessel disease burden, network disconnection, and vascular permeability are robust and interrelated predictors of post-stroke cognitive impairment, with some imaging markers demonstrating particularly large effect sizes, exceeding OR 9–12 for long-term outcomes.

Brain frailty was also consistently associated with poorer functional recovery following ischemic stroke and endovascular thrombectomy. Multiple studies demonstrated that greater burden of imaging-defined brain frailty markers, including white matter hyperintensities, cerebral atrophy, and composite brain frailty scores, was associated with reduced likelihood of achieving favorable functional outcomes. Hong et al. [33] reported that higher white matter hyperintensity burden was associated with lower odds of excellent functional recovery after acute ischemic stroke. Similarly, Loewen et al. [24] found that greater brain frailty was independently associated with reduced odds of achieving an excellent functional outcome following thrombolysis. Gwak et al. [38] demonstrated that increasing white matter hyperintensity burden was associated with poorer functional outcomes across both mild and moderate-to-severe ischemic stroke populations. Among patients undergoing endovascular thrombectomy, Benali et al. [43] reported an association between brain atrophy and worse functional recovery, while Fladt et al. [45] found that higher composite brain frailty scores were linked to poorer neurological recovery trajectories. Griessenauer et al. [44] similarly observed reduced odds of favorable 90-day functional outcomes with increasing white matter hyperintensity burden. The overall evidence suggests that structural markers of brain frailty are generally associated with impaired functional recovery after stroke (Table 5).

### 3.5. Risk of Bias Assessment

The included studies were generally of high methodological quality. Of the 15 included studies, 13 were classified as having a low risk of bias, with NOS scores ranging from 7 to 9 stars, whereas Munthe-Kaas et al. [34] and Fladt et al. [45] were classified as having a moderate risk of bias (6/9). Gwak et al. [38] achieved the maximum NOS score of 9 stars, reflecting high methodological quality across the selection, comparability, and outcome assessment domains.

The most frequently identified methodological concerns included uncertainty regarding baseline cognitive or outcome status, incomplete follow-up, missing outcome data, and incomplete reporting of baseline characteristics. Less common concerns included limited generalizability, highly selected study populations, and insufficient reporting of outcome assessment methods.

Methodological heterogeneity was also observed across the included studies. Baseline stroke severity differed considerably between study populations, with reported NIHSS scores ranging from minor stroke cohorts to patients with more severe stroke undergoing endovascular thrombectomy. In addition, adjustment strategies were not uniform across studies. Furthermore, cognitive outcomes were assessed using different outcome definitions and assessment methods, including post-stroke cognitive impairment, major or minor neurocognitive disorder, persistent cognitive impairment, and overall cognitive performance.

**Figure 2:**
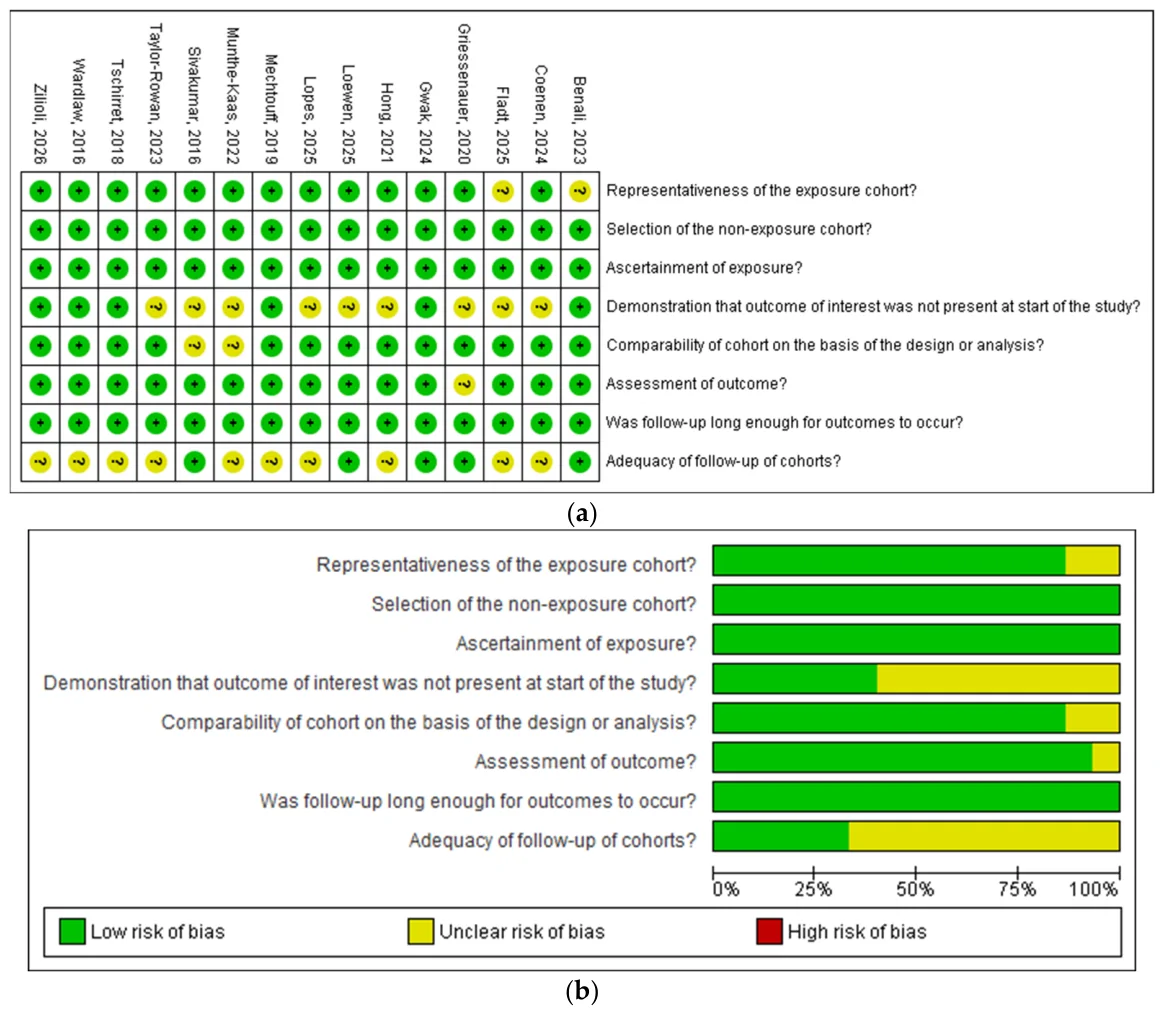
NOS risk-of-bias assessment generated in RevMan. (**a**) Traffic plot (**b**) Summary plot [24,25,33,34,35,36,37,38,39,40,41,42,43,44,45].

Overall, the NOS assessment suggests that the included studies were generally of good methodological quality and were associated with a predominantly low risk of bias. Nevertheless, the methodological differences identified across the included studies should be considered when comparing their findings and interpreting the overall body of evidence (Figure 2). The detailed NOS assessment for each included study is provided in the Supplementary Material (Table S3).

## 4. Discussion

This systematic review investigated the role of brain frailty in the outcomes of stroke patients—a topic of profound clinical relevance. Although the high heterogeneity among the included studies precluded a quantitative meta-analysis, our qualitative synthesis yields critical insights that underscore the necessity of integrating brain frailty assessments of stroke patients.

### 4.1. Post-Stroke Cognitive Impairment

The robust associations observed in this review between structural neuroimaging markers and post-stroke cognitive deficits strongly highlight the clinical utility of brain frailty indices in predicting long-term cognitive trajectories. Lopes et al. demonstrated that structural disconnection burden profoundly predicts post-stroke cognitive impairment at 6 months (OR = 9.96) and that this risk escalates further by 36 months (OR = 12.27) [35]. Similarly, Sivakumar et al. reported a nearly tenfold increase in the likelihood of persistent cognitive impairment among patients exhibiting greater burdens of MRI-defined small vessel disease markers (OR = 9.42) [42]. This longitudinal trajectory is mirrored by Munthe-Kaas et al., who identified a more than threefold increase in the risk of major neurocognitive disorder (OR = 3.09) as early as 3 months post-stroke due to medial temporal atrophy [34].

Several interrelated pathophysiological mechanisms may explain why baseline brain frailty so powerfully drives post-stroke cognitive decline. First, brain frailty markers—such as atrophy or pre-existing structural disconnection—signify a brain that has already lost significant neural architecture, which ultimately compromises cognitive function [20,21,22]. One of the most common markers used in our included studies is WMH, which appears to be a primary pathology in cognitive decline associated with brain atrophy [20,21,22]. Interestingly, a study by Wardlaw et al. highlighted that brain frailty often co-occurs with chronic, progressive pathologies like BBB leakage (β −3.62 to −0.16, *p* < 0.001).

### 4.2. Functional Outcome After Ischemic Stroke

Functional outcomes were also significantly influenced by brain frailty. A study by Hong et al. showed that higher WMH volumes were associated with a lower likelihood of achieving excellent outcomes (mRS 0–1) [33]. These diminished functional outcomes may be directly linked to the cumulative cognitive and structural decline driven by underlying brain frailty markers—such as baseline atrophy or pre-existing structural disconnection—which signify a brain that has already lost significant neural architecture [20,21,22]. Evaluating these baseline markers allows clinicians to identify high-risk patients much earlier, providing major implications for long-term prognosis.

### 4.3. Outcome After Thrombectomy or Thrombolysis

The negative prognostic impact of baseline cerebral changes remains evident even when patients receive advanced acute reperfusion therapies. When patients underwent thrombolysis, brain frailty was found to decrease the probability of an excellent outcome, as demonstrated in a study by Loewen et al. [24]. Similarly, evaluating advanced endovascular therapies, Fladt et al. reported that brain frailty markers were associated with poorer functional outcomes following EVT [45].

### 4.4. Hemorrhagic Stroke

While the clinical data compiled in this review heavily focus on ischemic stroke cohorts, the underlying structural and vascular vulnerabilities that define brain frailty likely manifest through distinct pathophysiological pathways when contrasting ischemic and hemorrhagic events. A study by Ong et al. on SICH patients showed that brain frailty was significantly associated with lower overall survival (HR: 1.54; 95% CI: 1.11–2.14, *p* = 0.010) and poor 90-day functional outcomes (OR: 1.90; 95% CI: 1.32–2.74; *p* < 0.001), even after adjusting for confounding variables [26]. Markers such as BBB leakage signify a brittle, unstable microvasculature. When a hemorrhagic breach occurs, this baseline vascular fragility compromises hematoma containment—escalating the risk of early hematoma expansion—while the compromised BBB leaves the surrounding brain parenchyma highly susceptible to the secondary neurotoxic effects of extravasated blood products. Studies investigating the relationship between hemorrhagic stroke and the frail brain are strongly warranted due to limited data in the literature.

### 4.5. Future Directions: The Role of Quantitative Susceptibility Mapping (QSM) in Brain Frailty

Quantitative susceptibility mapping (QSM) is a novel MRI technique that provides both anatomical and physiological information about brain tissue by measuring magnetic susceptibility [46]. It is highly sensitive to local and global oxygenation changes. A study by Uchida et al. [47] demonstrated that QSM can capture critical susceptibility-related tissue properties, including progressive iron deposition and myelin degradation. In cerebrovascular disease, longitudinal MRI studies have shown that increased magnetic susceptibility, accompanied by decreased R2* relaxometry values, within ischemic lesions reflects local iron-mediated neurotoxicity and demyelination.

In the context of brain frailty, baseline repair and clearance mechanisms are already compromised. Persistent iron-mediated neurotoxicity may impair remyelination, potentially explaining why patients with progressive iron accumulation experience poorer functional recovery.

Furthermore, QSM can quantify this vulnerability by estimating the oxygen extraction fraction (OEF) through measurements of venous deoxygenated hemoglobin [47]. QSM-derived OEF maps rapidly identify tissue under severe metabolic stress and have been shown to correspond closely with penumbral regions identified on dynamic susceptibility contrast MRI (DSC-MRI) [48]. For patients with a high degree of brain frailty, who possess a limited physiological reserve, early OEF mapping may help identify salvageable tissue before it progresses to irreversible infarction.

However, these proposed mechanisms remain hypothetical, and further studies are needed to clarify the relationship between QSM and brain frailty and to determine whether QSM can reliably identify imaging biomarkers of a frail brain. Future comprehensive brain frailty frameworks should therefore extend beyond macrostructural abnormalities and incorporate multiparametric QSM-derived markers. Integrating conventional structural metrics, such as WMHs and cerebral atrophy, with QSM-derived measures of iron burden, venous oxygenation, and OEF-related metabolic stress may facilitate the development of a more comprehensive, mechanistic imaging framework for assessing brain frailty.

### 4.6. Clinical Implications

Traditionally, post-stroke prognosis has relied heavily on acute stroke characteristics, such as initial infarct size and lesion location. However, incorporating baseline brain frailty metrics may offer clinicians a broader understanding of a patient’s pre-existing neural reserve and long-term recovery potential. For instance, patients with high WMH burdens or severe structural disconnection can be identified early as having a higher risk for post-stroke cognitive decline and incomplete functional recovery. Furthermore, the synthesized evidence suggests an association between higher baseline brain frailty and a lower likelihood of achieving an excellent functional outcome (mRS 0–1), even among patients receiving acute reperfusion therapies like intravenous thrombolysis or EVT. These findings highlight the potential value of brain frailty indices as prognostic tools to refine risk stratification. Future studies are needed to guide clinical decision-making and to identify and appropriately weight the most critical brain frailty and clinical factors that may modify the benefit–risk balance of reperfusion therapy.

Overall, confidence in the current evidence is considered moderate. Most included studies were of good methodological quality according to the Newcastle–Ottawa Scale; however, confidence is reduced by substantial clinical heterogeneity, variability in imaging definitions of brain frailty, inconsistent adjustment for important confounders such as infarct volume and baseline NIHSS, differences in cognitive outcome assessment, and the observational design of most included studies. Consequently, the findings should be interpreted as prognostic associations rather than evidence of causality.

### 4.7. Limitations

Several limitations should be acknowledged in this review. First, the substantial clinical and methodological heterogeneity across included studies rendered a quantitative meta-analysis unfeasible, requiring a narrative synthesis. Second, the included studies varied widely across neuroimaging modalities, brain frailty definitions, follow-up durations, and stroke subtypes. Specifically, the reliance on CT in some cohorts likely underestimated subtle vascular and structural frailty markers compared to advanced MRI techniques (e.g., DCE-MRI BBB leakage). Furthermore, disparate scoring criteria prevent direct quantitative comparisons, as equivalent frailty scores across different scales may not represent equal biological burden. Additionally, varying follow-up durations complicate direct comparisons: short-term functional outcomes may primarily reflect acute lesion burden, whereas longer follow-ups capture cumulative cognitive and functional decline, making cross-study effect sizes inherently distinct. Finally, stroke subtype heterogeneity plays a confounding role; small-vessel ischemic strokes share common pathophysiological mechanisms with microvascular frailty, which may artificially inflate associations compared to non-small-vessel or hemorrhagic phenotypes, the latter of which remain markedly underrepresented in the current literature.

## 5. Conclusions

In this review, brain frailty was consistently associated with a decline in cognitive function and poorer functional outcomes. However, the types of studies included and the substantial heterogeneity must be carefully considered, necessitating caution when interpreting these data. Future RCTs are required to provide more precise data regarding the exact role of brain frailty in stroke outcomes.

## Figures and Tables

**Table 1:** Characteristics of the Included Studies.

Study	Country	Study Design	Population	Sample Size	Mean Age	Female (%)	Brain Frailty Marker(s)	Imaging Modality	Follow-Up	Baseline NIHSS
Hong et al., 2021 [33]	International multicenter	Multicenter retrospective cohort	Acute ischemic stroke survivors	890	63.7 y	42.6%	White matter hyperintensity (WMH) burden	MRI FLAIR	3–6 months	3
Taylor-Rowan et al., 2023 [25]	UK and Netherlands	Prospective longitudinal cohort	Stroke/TIA survivors	341	NR	NR	Leukoaraiosis, cerebral atrophy, old infarcts	CT brain	18 months	NR
Loewen et al., 2025 [24]	Canada	Post hoc RCT analysis	AIS thrombolysis patients	1568	74 y	47.9%	WMH, cortical atrophy, subcortical atrophy, chronic infarcts, lacunes	NCCT	90 days	9
Munthe-Kaas et al., 2022 [34]	Norway	Prospective cohort	Stroke survivors	598	71.6 y	43.0%	Medial temporal atrophy	CT/MRI	3 months	3
Lopes et al., 2025 [35]	France	Prospective longitudinal cohort	First-ever minor ischemic stroke	105 analyzed	63 ± 12 y	31%	WMH volume, infarct location, tract disconnection burden	MRI	36 months	1
Zilioli et al., 2026 [36]	Italy	Retrospective cohort	AIS patients	450	73.6 ± 14.0 y	53.8%	Regional atrophy, WML burden, API	NCCT	90 days	14
Coenen et al., 2024 [37]	International multicenter	Pooled cohort analysis	Ischemic stroke patients	1568	67.3 y	39.9%	Regional WMH tract-specific burden	MRI	3–6 months	3.5
Gwak et al., 2024 [38]	South Korea	Registry analysis	AIS patients	8918	67.2 ± 12.6 y	39.9%	WMH volume and infarct volume	MRI	90 days–1 year	4
Wardlaw et al., 2017 [39]	United Kingdom	Prospective cohort	Mild ischemic stroke	170	67 y	NR	Blood–brain barrier leakage	DCE-MRI	12 months	2
Tschirret et al., 2018 [40]	France	Prospective cohort	Severe AIS	137	72.5 y	47.4%	Relative cerebral volume (atrophy)	CT/MRI	90 days	19
Mechtouff et al., 2019 [41]	France	Retrospective cohort	Mechanical thrombectomy patients	293	67.1 y	45.4%	WMH burden	MRI	90 days	15.5
Sivakumar et al., 2016 [42]	Canada	Prospective cohort	TIA/minor stroke	115	66 y	35%	DWI lesion volume, lacunes, microbleeds	MRI	90 days	3
Benali et al., 2023 [43]	Multinational	Post hoc RCT analysis	EVT-treated AIS	1102	69.5 ± 13.7 y	~43%	Cortical, hippocampal, parietal and subcortical atrophy	CT/MRI	90 days	≥6
Griessenauer et al., 2020 [44]	United States	Retrospective cohort	AIS patients	1433	67.7 ± 13.4 y	49.2%	WMH volume	MRI/CTA	90 days	3
Fladt et al., 2025 [45]	Multinational	Post hoc RCT analysis	EVT-treated AIS	1102	69.5 y	49.7%	Cortical atrophy, subcortical atrophy, WMH, chronic infarcts	NCCT/CTA	90 days	≥5

Abbreviations: AIS, acute ischemic stroke; CTA, computed tomography angiography; CT, computed tomography; DCE-MRI, dynamic contrast-enhanced magnetic resonance imaging; EVT, endovascular thrombectomy; MRI, magnetic resonance imaging; NCCT, non-contrast computed tomography; NR, not reported; NIHSS, National Institutes of Health Stroke Scale; TIA, transient ischemic attack; WMH, white matter hyperintensity.

**Table 2:** Brain Frailty Components and Scoring Systems.

Study	Brain Frailty Components	Assessment Method
Hong 2021 [33]	WMH burden	Automated WMH segmentation
Taylor-Rowan 2023 [25]	Leukoaraiosis + cerebral atrophy + old infarcts	Visual CT-based brain frailty score (0–3)
Loewen 2025 [24]	Fazekas + GCA + CC:IT + chronic infarcts/lacunes	Composite CT brain frailty score
Munthe-Kaas 2022 [34]	Medial temporal atrophy	MTA scale
Lopes 2025 [35]	Structural disconnection burden	Bayesian LDA model
Zilioli 2026 [36]	MTA + GCA-F + Koedam + Fazekas	Visual rating scales
Coenen 2024 [37]	Strategic WMH tract burden	MRI tract mapping
Gwak 2024 [38]	WMH volume quintiles	Automated volumetric analysis
Wardlaw 2017 [39]	BBB leakage	DCE-MRI permeability mapping
Tschirret 2018 [40]	Brain atrophy	Relative cerebral volume
Mechtouff 2019 [41]	WMH burden	Fazekas score
Sivakumar 2016 [42]	DWI lesions + lacunes + microbleeds	MRI markers of SVD
Benali 2023 [43]	Cortical, hippocampal, parietal, subcortical atrophy	Visual CT/MRI assessment
Griessenauer 2020 [44]	WMH volume	Quantitative MRI
Fladt 2025 [45]	Atrophy + WMH + chronic infarcts	Composite brain frailty score

Abbreviations: BBB, blood–brain barrier; CC:IT, central cerebral atrophy to inner table ratio; CT, computed tomography; DCE-MRI, dynamic contrast-enhanced magnetic resonance imaging; GCA, global cortical atrophy; GCA-F, global cortical atrophy—frontal; LDA, latent Dirichlet allocation; MRI, magnetic resonance imaging; MTA, medial temporal atrophy; SVD, small vessel disease; WMH, white matter hyperintensity.

**Table 3:** Operational Domains of Brain Frailty.

Domain	Representative Markers	Included Studies
Chronic CSVD burden	WMH, lacunes, chronic infarcts, enlarged perivascular spaces	Hong, Gwak, Mechtouff, Griessenauer [33,38,41,44]
Neurodegenerative changes	Cortical atrophy, subcortical atrophy, medial temporal atrophy, hippocampal atrophy	Munthe-Kaas, Zilioli, Benali, Fladt [34,36,43,45]
Network injury	Structural disconnection burden, strategic WMH tract involvement	Lopes, Coenen [35,37]
Physiological vascular dysfunction	BBB leakage, permeability imaging	Wardlaw [39]

**Table 4:** Primary Outcomes Evaluated.

Study	Primary Outcome
Hong 2021 [33]	Functional outcome (mRS)
Taylor -Rowan 2023 [25]	Major/minor neurocognitive disorder
Loewen 2025 [24]	Excellent functional outcome (mRS 0–1)
Munthe-Kaas 2022 [34]	Post-stroke neurocognitive disorder
Lopes 2025 [35]	Post-stroke cognitive impairment
Zilioli 2026 [36]	Futile recanalization
Coenen 2024 [37]	Cognitive impairment
Gwak 2024 [38]	Poor functional recovery
Wardlaw 2017 [39]	Cognitive performance
Tschirret 2018 [40]	Early neurological deterioration
Mechtouff 2019 [41]	Thrombectomy outcome
Sivakumar 2016 [42]	Persistent cognitive impairment
Benali 2023 [43]	Ordinal mRS shift
Griessenauer 2020 [44]	90-day functional outcome
Fladt 2025 [45]	Neurologic recovery and NIHSS trajectory

Abbreviations: mRS, modified Rankin Scale; NIHSS, National Institutes of Health Stroke Scale.

**Table 5:** Adjusted Effect Estimates for the Association Between Brain Frailty Markers and Clinical Outcomes.

Study	Outcome	Effect Estimate	95% CI	*p* Value
Hong et al., 2021 [33]	Poor functional outcome after acute ischemic stroke	Higher WMH burden associated with lower odds of excellent outcome (mRS 0–1)	Not fully reported; significant reduction in odds observed	<0.01
Taylor-Rowan 2023 [25]	Cognitive impairment and frailty outcomes	Brain Frailty OR = 1.64	1.17–2.32	<0.001
		Brain Frailty HR = 1.84	1.29–2.62	<0.001
		Rockwood Frailty OR = 1.05	1.02–1.08	<0.001
		Fried Frailty OR = 1.93	1.39–2.67	<0.001
Loewen et al., 2025 [24]	Excellent functional outcome after thrombolysis	Adjusted OR = 0.40	0.24–0.65	<0.001
Munthe-Kaas et al., 2022 [34]	Major neurocognitive disorder at 3 months	Adjusted OR = 3.09	NR	NR
Lopes et al., 2025 [35]	Post-stroke cognitive impairment at 6 months	OR = 9.96	2.21–52.40	0.020
	Post-stroke cognitive impairment at 36 months	OR = 12.27	2.80–63.40	0.006
Zilioli et al., 2026 [36]	Futile recanalization after EVT	OR ≈ 2.19	1.32–3.63	0.002
Coenen et al., 2024 [37]	Post-stroke cognitive impairment	Significant association between strategic WMH location and cognitive impairment	Reported as significant	<0.05
Gwak et al., 2024 [38]	Poor outcome in mild ischemic stroke	OR = 1.89	NR	<0.001
	Poor outcome in moderate-to-severe ischemic stroke	OR = 1.69	NR	<0.001
Wardlaw et al., 2017 [39]	Cognitive decline associated with BBB leakage	β coefficient significant	−3.62 to −0.16	<0.001
Tschirret et al., 2018 [40]	Early neurological deterioration and functional outcome	Significant association between brain atrophy and outcome	NR	<0.05
Mechtouff et al., 2019 [41]	Functional outcome after thrombectomy	OR ≈ 0.90	0.54–1.49	0.66
Sivakumar et al., 2016 [42]	Persistent cognitive impairment	OR ≈ 9.42	1.956–45.369	0.005
Benali et al., 2023 [43]	Functional recovery after EVT	OR ≈ 0.65	0.45–0.94	0.003
Griessenauer et al., 2020 [44]	Favorable 90-day functional outcome	OR = 0.863	0.767–0.972	<0.05
Fladt et al., 2025 [45]	Neurological recovery after EVT	Significant association between composite brain frailty score and poorer recovery	NR	<0.05

Abbreviations: BBB, blood–brain barrier; CI, confidence interval; EVT, endovascular thrombectomy; HR, hazard ratio; mRS, modified Rankin Scale; NR, not reported; OR, odds ratio; WMH, white matter hyperintensity; β, beta coefficient.

## Data Availability

No new data were created or analyzed in this study. Data sharing is not applicable to this article.

## Supplementary Materials

The following supporting information can be downloaded at https://www.mdpi.com/article/10.3390/neurolint18080147/s1. Table S1: Detailed Search Strategies Used Across Databases, Table S2: The Preferred Reporting Items for Systematic Reviews (PRISMA) checklist, Table S3: Detailed Newcastle-Ottawa Scale (NOS) Assessment for the Included Cohort Prospective and Retrospective Studies.
